# Impact of Chemotherapy-Induced Peripheral Neuropathy on Quality of Life in Patients with Advanced Lung Cancer Receiving Platinum-Based Chemotherapy

**DOI:** 10.3390/ijerph18115677

**Published:** 2021-05-26

**Authors:** Hsing-Wei Hung, Chien-Ying Liu, Hsiu-Fang Chen, Chun-Chu Chang, Shu-Ching Chen

**Affiliations:** 1 Department of Nursing, Chang Gung Memorial Hospital at Linkou, Taoyuan 333, Taiwan; hsingwei@cgmh.org.tw (H.-W.H.); fang@gw.cgust.edu.tw (H.-F.C.); m22096@cgmh.org.tw (C.-C.C.); 2 School of Nursing, College of Medicine, Chang Gung University, Taoyuan 333, Taiwan; 3 Division of Thoracic Oncology, Department of Thoracic Medicine, Chang Gung Memorial Hospital at Linkou, Taoyuan 333, Taiwan; cyliu01@cgmh.org.tw; 4 Thoracic Oncology Unit, Cancer Center, Chang Gung Memorial Hospital at Linkou, Taoyuan 333, Taiwan; 5 Department of Medicine, College of Medicine, Chang Gung University, Taoyuan 333, Taiwan; 6 Department of Nursing, College of Nursing, Chang Gung University of Science and Technology, Taoyuan 333, Taiwan; 7 School of Nursing and Geriatric and Long-Term Care Research Center, College of Nursing, Chang Gung University of Science and Technology, Taoyuan 333, Taiwan; 8 Department of Radiation Oncology and Proton and Radiation Therapy Center, Linkou Chang Gung Memorial Hospital, Taoyuan 333, Taiwan

**Keywords:** lung cancer, chemotherapy, cisplatin, carboplatin, chemotherapy-induced peripheral neuropathy, quality of life

## Abstract

Chemotherapy-induced peripheral neuropathy (CIPN) is a common adverse effect of neurotoxic anticancer drugs that may affect quality of life (QoL). Purpose: The purposes of this study were to: assess the levels of CIPN, anxiety, depression, CIPN–related QoL, and general QoL; and identify the factors related to CIPN–related QoL and general QoL in patients with advanced lung cancer (LC) receiving platinum-based chemotherapy. This cross-sectional study examined patients with advanced LC who received platinum-based chemotherapy from the thoracic oncology inpatient wards of a medical center in northern Taiwan. Structured questionnaires were used to measure patients’ CIPN (European Organization for Research and Treatment of Cancer quality of life questionnaire–chemotherapy–induced peripheral neuropathy 20), anxiety (Hospital Anxiety and Depression Scale Depression Scale [HADS]), depression (HADS), CIPN-related QoL (Functional Assessment of Cancer Therapy /Gynecologic Oncology Group-Neurotoxicity subscale [FACT/GOG–Ntx]), and general QoL (Functional Assessment of Cancer Therapy–General Input [FACT-G]). Of 93 patients with advanced LC, 53.8% reported CIPN–sensory impairment and 47.3% reported CIPN–motor impairment. The most common CIPN symptoms were difficulty getting or maintaining an erection (only for men > 65 years) and difficulty in climbing stairs or getting up out of a chair. Poor CIPN–related QoL (FACT/GOG–Ntx) was associated with more CIPN–sensory and more CIPN–motor impairment. Poor general QoL (FACT-G) was associated with a higher level of depression, a higher level of anxiety, and receipt of more chemotherapy cycles. More than half of LC patients report impairment related to CIPN, calling for holistic treatment to improve QoL.

## 1. Introduction

Lung cancer (LC) is the most common cancer worldwide, with an estimated 2.1 million new cases and approximately 1.76 million deaths expected annually [1]. In Taiwan, some 12,000 new LC cases are reported per year [2]. About 75% of patients are diagnosed at an advanced stage, and chemotherapy is the major treatment modality [3]. Chemotherapy-induced peripheral neuropathy (CIPN) is a common adverse effect of neurotoxic anticancer drugs, including platinum-based agents, such as cisplatin and carboplatin [4]. Approximately 70% of patients treated with platinum-based antineoplastics report CIPN [4], which may develop 1–2 weeks after initiating treatment, persist for several months after completing treatment, and depend on the cumulative dose exposure [5]. Very limited options exist for the treatment of CIPN [6].

CIPN refers to motor, sensory, and autonomic neurons dysfunction, which presents as peripheral neuropathic signs and symptoms, and includes sensory damage. The predominant symptoms of CIPN are paresthesia, numbness and tingling, dulled sensations in the peripheral nerves, burning and shooting pain, or electric shock–like pain [3,7]; motor damage can be manifested as weakness, gait and balance disturbance, and difficulty with fine motor skills [7]. Autonomic damage dysfunction presents as constipation, orthostatic hypotension, and urinary incontinence [8]. Albany et al. [9] found that patients who received cisplatin regimens report neuropathy symptoms more similar to those of patients receiving oxaliplatin than for those receiving paclitaxel. Cisplatin-induced neuropathy was less severe than that seen in patients who received oxaliplatin or a combination of doxorubicin and cyclophosphamide, potentially because the cisplatin-receiving patients were younger. Le-Rademacher et al. [10] reported that patients received oxaliplatin and paclitaxel/carboplatin regimens had chemotherapy-induced peripheral neuropathy, with European Organization for Research and Treatment of Cancer quality of life questionnaire–chemotherapy–induced peripheral neuropathy 20 (EORTC QLQ-CIPN20) scores and US National Cancer Institute Common Terminology Criteria for Adverse Events 4.03v (NCI-CTCAE) grades being strongly positively correlated. Ezzi et al. [11] also showed that, of patients who underwent chemotherapy with cisplatin for at least 2 months at Kenyatta National Hospital oncology units, 56 patients (83.6%) had peripheral neuropathy; 45 patients (81%) had mild-grade (grades 1 and 2) peripheral neuropathy and only two patients (3.1%) had grade 4 neuropathy. The neuropathy symptoms may cause dose reduction [12], refusal to continue treatment [13], psychological distress [14], neurological impairment [15], and reduced quality of life (QoL) [16,17].

Although previous studies have explored these issues, most research has focused on patients with colorectal cancer [18,19,20], breast cancer [21], or those in Western countries [22,23,24]; or on other chemotherapy regimens such as taxane [22,23], paclitaxel [23,24], or carboplatin [23,24]. Clinical observation indicates that LC patients treated with platinum-based regimens report CIPN-related symptoms that affect daily function and QoL, and insufficient attention has been devoted to this important topic. Therefore, the purposes of this study were to (1) examine the levels of CIPN, anxiety, depression, general QoL, and CIPN–related QoL; and (2) identify the factors associated with CIPN–related QoL and general QoL among advanced LC patients receiving platinum-based chemotherapy.

## 2. Methods

### 2.1. Design

A descriptive cross-sectional study was conducted with a convenience sample from the thoracic oncology inpatient wards of a medical center in northern Taiwan. Data were collected from September 2017 through June 2019.

### 2.2. Sample

The inclusion criteria were: (1) diagnosis of stage III B or IV LC; (2) receiving platinum-based chemotherapy with cisplatin or carboplatin regimens; (3) receiving at least four cycles of chemotherapy; (4) agreement to participate in the study after explanation of its purposes and procedures; and (5) aged 20 years or older. Patients were not eligible if they had a mental disorder, hypothyroidism, alcoholism, vitamin B1 or B12 insufficiency, discontinuation of scheduled treatment more than two months since prior treatment cycles, other comorbid conditions potentially causing CINP (diabetes, thyroid disease, or preexisting neuropathy), or a physical performance less than 60 on the Karnofsky Performance Status Scale (KPS) [25].

The systemic therapy protocol for advanced or metastatic disease of non-small cell LC was as follows (beyond of 4–6 cycles) [26]: carboplatin/albumin-bound paclitaxel; carboplatin/docetaxel; carboplatin/gemcitabine; carboplatin/paclitaxel; cisplatin/docetaxel; cisplatin/etoposide; cisplatin/gemcitabine; cisplatin/paclitaxel; cisplatin/docetaxel; or cisplatin/vinorelbine.

The chemotherapy protocol for extensive stage small cell LC was as follows (maximum of 4–6 cycles) [27]: carboplatin AUC 5–6 day 1 and etoposide 100 mg/m^2^ days 1,2,3; cisplatin 75 mg/m^2^ day 1 and etoposide 100 mg/m^2^ days 1,2,3; cisplatin 80 mg/m^2^ day 1 and etoposide 80 mg/m^2^ days 1,2,3; cisplatin 25 mg/m^2^ day 1,2,3 and etoposide 100 mg/m^2^ days 1,2,3; carboplatin AUC 5 day 1 and irinotecan 60 mg/m^2^ days 1,8,15; cisplatin 60 mg/m^2^ day 1 and irinotecan 60 mg/m^2^ days 1,8,15; or cisplatin 30 mg/m^2^ day 1,8 and irinotecan 65 mg/m^2^ days 1,8.

### 2.3. Ethical Considerations

Ethical approval was obtained from the Institutional Review Board of the study institution (Number: 201701101B0). The study was conducted in accordance with the Declaration of Helsinki, and written informed consent was obtained from all participants after the study goals and procedures were explained to each in detail.

### 2.4. Data Collection

LC patients who met the inclusion criteria were contacted by a research nurse. Participants filled out the structured questionnaires by self-report in the wards; when necessary, the research nurse read out each item of the questionnaire, which took around 10–15 min.

### 2.5. Measures

#### 2.5.1. European Organization for Research and Treatment of Cancer Quality of Life Questionnaire–Chemotherapy–Induced Peripheral Neuropathy 20 (EORTC QLQ-CIPN20)

CIPN was assessed using the EORTC QLQ-CIPN20 [28]. The 20-item scale includes three subscales: sensory (9 items), motor (8 items), and autonomic (3 items). Each item is scored from 1 (not at all) to 4 (very much). The summed scores of each subscale and the overall CIPN scale are converted into standardized scores ranging from 0 to 100, with higher scores representing more CIPN–related symptom distress. The scale was translated into Chinese, and previous cancer-related studies reported satisfactory psychometric properties for the EORTC QLQ–CIPN20 [18,21]. The EORTC QLQ–CIPN20 has been demonstrated to be reliable in a cancer-related study [29]. The Cronbach’s α for this study was 0.88.

#### 2.5.2. Hospital Anxiety and Depression Scale Depression Scale (HADS)

Anxiety and depression were assessed using the Hospital Anxiety and Depression Scale (HADS) [25]. Each of the 14 items (7 for anxiety and 7 for depression) were scored from 0 (not at all) to 3 (always) and the total score ranges from 0 to 21, with a higher score indicating greater anxiety or depression. Scores of 0 to 7 indicate the absence of anxiety or depression; scores of 8 to 10 indicate borderline anxiety or depression; and scores of 11 to 21 indicate clinical anxiety or depression [30]. The scale was translated into Chinese, and a previous cancer-related study reported satisfactory psychometric properties for the HADS [31]. Cronbach’s alpha for the HADS-Anxiety Subscale and HADS-Depression Subscale in the present study was 0.90 and 0.88, respectively.

#### 2.5.3. Functional Assessment of Cancer Therapy/Gynecologic Oncology Group-Neurotoxicity Subscale (FACT/GOG–Ntx)

The CIPN-related QoL was assessed using the Functional Assessment of Cancer Therapy/Gynecologic Oncology Group-Neurotoxicity subscale (FACT/GOG–Ntx) [32]. The instrument consists of 11 items, with responses scored on a Likert scale from 0 (not at all) to 4 (very much). The summed scores of each item were reverted into standardized scores ranging from 0 to 44, with a higher score indicating a lower level of neurological toxicity and less effect on QoL. Satisfactory psychometric properties were reported in a previous study [32]. Cronbach’s alpha for the FACT/GOG–Ntx was 0.90.

#### 2.5.4. Functional Assessment of Cancer Therapy–General (FACT–G)

The Functional Assessment of Cancer Therapy-General (FACT–G) was used to assess general QoL in patients with LC [33]. The 27 item FACT–G contains 5 domains: physical well-being (PWB) (7 items), social/family well-being (SWB) (7 items), emotional well-being (EWB) (6 items), and functional well-being (FWB) (7 items) [27]. Each item is scored on a scale of 0 to 4, with a higher score indicating a better QoL. The FACT–G has been demonstrated to be reliable in a cancer-related study [34]. In the present study, the Cronbach’s α was 0.88.

#### 2.5.5. The National Cancer Institute Common Terminology Criteria for Adverse Events 4.03v (NCI-CTCAE)

The US NCI-CTCAE 4.03v is widely used in oncology care as the standard classification and severity grading scale for adverse events in cancer therapy clinical trials and other oncology settings [35]. Severity of peripheral motor neuropathy or peripheral sensory impairment is assessed in 5 grades, ranging from 0 to 5: Grade 1, Asymptomatic, clinical or diagnostic observations only; intervention not indicated/loss of deep tendon reflexes or paresthesia; Grade 2, Moderate symptoms; limiting instrumental activities of daily living (ADL); Grade 3, Severe symptoms; limiting self-care ADL; assistive device indicated; Grade 4, Life-threatening consequences; urgent intervention indicated; and Grade 5, Death [35]. The inter-rater reliability for this study was 0.99 between the research nurse and the oncologist who provided training to the research nurse.

#### 2.5.6. Karnofsky Performance Status Scale (KPS Scale)

The KPS scale, used to assess a patient’s performance status, ranges from 100% (normal function) to 0% (death) [24]. The KPS has been used in clinical cancer studies to assess cancer patients’ level of physical function [36,37]. An inter-observer reliability coefficient of 0.98 was reported in the present study.

#### 2.5.7. Demographic and Clinical Characteristics Form

Demographic data (age, gender, occupation, marital status, education level, and religion) and characteristics of the cancer and treatment (histology type, cancer stage, number of chemotherapy cycles, chemotherapy modalities, total chemotherapy dose, time since diagnosis, and time since the completion of previous chemotherapy) were extracted from the medical record.

### 2.6. Statistical Methods

SPSS, version 26.0 for Windows (IBM Corp., Armonk, NY, USA) was used to analyze the data. Descriptive statistics (frequency distribution, percentage, means, standard deviation errors [SE]) were used to analyze the demographic and clinical characteristics; and the levels of CIPN, depression, CIPN–related QoL, and general QoL. Multiple stepwise regression was used to identify factors associated with CIPN–related QoL and general QoL. The independent variables included marital status, number of chemotherapy cycles, cumulative chemotherapy dose, the EORTC QLQ-CIPN20 scores (CIPN–sensory score, CIPN–motor score, CIPN–autonomic score), anxiety, and depression.

## 3. Results

### 3.1. Demographic and Clinical Characteristics

Of 100 eligible patients approached, 7 declined to participate because they had no time or interest; from a total of 93 patients in the study, 93.0% of all patients were approached. Patients’ average age was 59.24 ± 1.20 years. The majority were male (*n* = 54, 58.1%), unemployed (*n* = 66, 71.0%), married (*n* = 65, 69.9%), had a junior high school education or less (*n* = 48, 51.7%), and held Buddhist/Taoist religious beliefs (*n* = 62, 66.7%). A majority of patients were diagnosed with non-small cell LC (*n* = 74, 79.6%) and stage IV disease (*n* = 76, 81.7%). The average number of chemotherapy cycles was 5.53; most patients received four cycles of chemotherapy (*n* = 45, 48.4%); the most common regimen was cisplatin (*n* = 70, 75.3%); the mean total platinum dose was 682.94 mg (standard error of the mean [SE] = 49.57, range: 121 to 2756 mg); and all patients had satisfactory KPS scores (60–100). CIPN assessed using the NCI-CTCAE reported that a total of 53.8% (*n* = 50) of the study subjects had CIPN–sensory impairment with toxicity grade I (36.6%, *n* = 34) or grade II (17.2%, *n* = 16), and a total of 47.3% (*n* = 44) had CIPN–motor impairment with toxicity grade I (32.3%, *n* = 30) or grade II (15.0%, *n* = 14). The rest of the patients reported no CIPN–sensory (46.2%, *n* = 43) or CIPN–motor (52.7%, *n* = 49) impairment. The average time since diagnosis was 10.67 months and the average time since the completion of previous chemotherapy was 4.28 weeks (Table 1).

### 3.2. Top Chemotherapy-Induced Peripheral Neuropathy (CIPN) Symptoms

The highest-scoring CIPN symptoms in descending ranking were: “did you have difficulty getting or maintaining an erection?” (mean = 56.02, SE = 4.33), this item only for men < 65 years (*n* = 37); “did you have difficulty climbing stairs or getting up out of a chair because of weakness in your legs?” (mean = 40.59, SE = 2.02); and “did you have blurred vision?” (mean = 37.63, SE = 1.56) (Table 2).

### 3.3. Levels of CIPN, Depression, CIPN–Related Quality of Life (QoL), and General QoL

The overall QLQ–CIPN 20 score was 31.56 (SE = 0.84). Mean scores for the subscales were: sensory, 29.15 (SE = 0.57); motor, 31.83 (SE = 0.93); and autonomic, 33.81 (SE = 1.45). The mean score for anxiety was 3.74 (SE = 0.41) and that for depression was 6.67 (SE = 0.49). According to the HADS classification, 6.5% (*n* = 6) and 7.5% (*n* = 7) of patients were classified as having clinical anxiety or borderline anxiety, respectively. According to the HADS classification, 23.7% (*n* = 22) and 17.2% (*n* = 16) of patients were classified as having clinical depression or borderline depression, respectively. The mean score for FACT/GOG–Ntx was 40.44 (SE = 0.57). The mean score for overall FACT–G was 78.08 (SE = 1.68). Mean scores for the subscales were: PWB, 20.79 (SE = 0.59); SFWB, 20.31 (SE = 0.43); EWB, 19.01 (SD = 0.50); and FWB, 17.97 (SE = 0.68) (Table 3).

### 3.4. Factors Associated with CIPN–Related QOL and General QoL

Multivariable analysis identified the factors that were associated with CIPN–related QoL (FACT/GOG–Ntx) and general QoL (FACT-G). The independent variables included marital status, number of chemotherapy cycles, cumulative chemotherapy dose, the EORTC QLQ-CIPN20 scores (CIPN–sensory score, CIPN–motor score, CIPN–autonomic score), anxiety, and depression. Patients who had greater CIPN–sensory scores (β = −0.651) and CIPN–motor scores (β = −0.263) were more likely to have worse CIPN–related QoL (FACT/GOG–Ntx). These two factors explained 64.5% of the total variance in CIPN-related QoL (FACT/GOG–Ntx). Patients who had a higher level of depression (β = −0.619), who had a higher level of anxiety (β = −323), and who received more chemotherapy cycles (β = 0.147) were more likely to have worse general QoL (FACT-G). These three factors explained 75.6% of the total variance in general QoL (FACT-G) (Table 4).

## 4. Discussion

In this study of patients with advanced LC who received platinum-based regimens, the prevalence of grade 0, I, II, and III CIPN–sensory adverse events were 46.2% (*n* = 43), 36.6% (*n* = 34), and 17.2% (*n* = 16), and 0% (*n* = 0), respectively, and the prevalence of grade 0, I, II, and III CIPN–motor adverse events were 52.7% (*n* = 49), 32.3% (*n* = 30), 15.0% (*n* = 14), and 0% (*n* = 0), respectively. Kautio et al. [38] found that, in LC patients who received vinca alkaloid, taxane, or platina derivative regimens, 0%, 21%, 42%, and 37% of patients reported a prevalence of grade 0, I, II, and III sensory CIPN adverse events, respectively, and 52%, 31%, 16%, and 1% of patients reported the prevalence of grade 0, I, II, and III motor CIPN adverse events, respectively. The reduced level of neuropathy may result from the fact that patients in Kautio et al. [38] had received 1–3 cycles of treatment, while our subjects had received at least 4 cycles of chemotherapy; indeed, more than half (51.6%) had received 5 or more cycles of treatment, and were still continuing treatment. CIPN symptoms were present within a few days after starting therapy and lasted for several months after treatment began. Perhaps the coasting effect was associated with platinum derivatives, with an appearance of CIPN or CIPN worsening after the end of the chemotherapy. These findings may reflect the timing of CIPN assessment, which may influence the severity and prevalence of CIPN. Healthcare providers should seek to detect CIPN early and continue assessment of CIPN as patients continue their chemotherapy.

Our results showed that the average CIPN–related QoL score of patients with advanced LC was 40.44 (SE = 0.57). The level of CIPN–related QoL among subjects in this study was lower than that reported in another recent study [39]. This difference may be due to the different cycles of chemotherapy during which patients were assessed. Patients in our study received an average of 5.53 cycles of chemotherapy and had not yet completed all cycles of chemotherapy, while patients in the study of Ajewole et al. [39] were enrolled within two weeks of the first chemotherapy of cycle 1 and followed for 12 weeks after enrollment. These findings may reflect an increase over time of the impact of CIPN on QoL. CIPN in advanced LC patients treated with platinum-based chemotherapy may impact their ability to perform self-care, independently complete ADLs, and return to work, as well as their level of physical activity. Healthcare providers should be aware of patients’ reports of outcomes related to CIPN and provide care for their needs.

The most highly rated CIPN items for patients in the present study were: difficulty getting/maintaining an erection, difficulty climbing stairs/rising from a chair, blurred vision, dizziness when standing up from a sitting/lying position, and numbness in the toes/feet. These findings agree with those of previous studies of cancer patients treated with CIPN-related regimens, which indicated that tingling or numbness in the fingers/hands, toes/feet were most the common CIPN–related symptoms, and may limit ADLs and diminish QoL [40,41]. In the present study, difficulty getting/maintaining an erection was the most common problem. Male patients may be embarrassed to discuss their perceptions and feelings about sexual dysfunction. Healthcare providers should therefore actively evaluate each patient’s concerns about changes in sexual function and encourage male patients in particular to express their feelings.

Results of the present study indicated that patients who had a higher level of depression and who had more severe CIPN–sensory scores were more likely to have worse overall QoL. These results are consistent with those of previous studies [42], which reported that CIPN [41] and depression [42] lead to restrictions in ADLs [42] and decreases in mobility and independence [43] in cancer patients treated with platinum-based antineoplastic agents. Healthcare professionals should evaluate neurological function and provide information on nutritional supplements, aerobic exercise, and balance training that may stimulate peripheral neurological function and enhance daily function, to help patients cope with the adverse effects of chemotherapy [44].

## 5. Limitations

This study had several limitations. First, the present study examined patients with advanced LC who received at least four chemotherapy cycles and, although such problems may develop as early as 1–2 weeks after initiating treatment, CIPN following chemotherapy cycles is part of a dynamic process that changes over time. Longitudinal studies are needed, with long-term follow up of patients from the time they undergo treatment through the post-treatment period. Second, we did not consider the effect of nutritional supplements (glutathione, glutamine, vitamin E, or vitamin B complex) or dietary supplements (vitamins, minerals, proteins, or amino acids) as factors in CIPN, although such factors may affect outcomes. Thus, future studies should extend their analysis to include the use of nutritional supplements [45,46,47]. Finally, patients’ initial (pre-treatment) peripheral neurological function and lifestyle, variables not studied here, may have affected the evaluation of CIPN. Further studies are needed to determine the correlation between initial peripheral neurological function, lifestyle, and CIPN [48].

## 6. Conclusions and Clinical Implications

### 6.1. Conclusions

We found that 53.8% and 47.3% of advanced LC patients receiving platinum-based chemotherapy reported CIPN–sensory impairment and CIPN–motor impairment, respectively. Patients who had greater CIPN–sensory and CIPN–motor impairment were more likely to have worse CIPN–related QoL (FACT/GOG–Ntx). Patients who had a higher level of depression who had a higher level of anxiety, and those receiving more chemotherapy cycles were more likely to have worse general QoL (FACT-G).

### 6.2. Clinical Implications

The results of this study provide a reference for clinical assessment of CIPN and the factors associated with CIPN–related QoL and general QoL in patients with advanced LC receiving platinum-based chemotherapy. Based on the results of the current study, these patients need a holistic approach to help them maximize their QoL, including health education, dietary guidance, nutritional supplements, aerobic exercise, and balance training.

## Figures and Tables

**Table 1:** Demographic and clinical characteristics of patients (*N* = 93).

Variable	Number (%)	Mean (SE)	Range
Age (years)		59.24(1.20)	27–89
Sex			
Male	54(58.1)		
Female	39(41.9)		
Occupation			
Unemployed	66(71.0)		
Employed	27(29.0)		
Marital status			
Unmarried	28(30.1)		
Married	65(69.9)		
Education level			
None	4(4.3)		
Elementary	18(19.4)		
Junior high	26(28.0)		
Senior high	28(30.1)		
College and above	17(18.2)		
Religion			
None	27(29.0)		
Buddhism/Taoism	62(66.7)		
Christianity/Catholicism	4(4.3)		
Histology type			
NSCLC	74(79.6)		
SCLC	19(20.4)		
Cancer stage			
III B	17(18.3)		
IV	76(81.7)		
Number of chemotherapy cycle	5.53(2.75)		4–22
4	45(48.4)		
5	24(25.8)		
≥6	24(25.8)		
Chemotherapy modalities			
Cisplatin	70(75.3)		
Carboplatin	5(5.4)		
Cisplatin + docetaxel	15(16.1)		
Carboplatin + docetaxel	3(3.2)		
Chemotherapy, total dose, mg/m^2^		682.94(49.57)	121–2756
Severity of CIPN–sensory impairment			
No CIPN impairment	43(46.2)		
NCI-CTCAE grade			
I	34(36.6)		
II	16(17.2)		
III	0(0)		
Severity of CIPN–motor impairment			
No CIPN impairment	49(52.7)		
NCI-CTCAE grade			
I	30(32.3)		
II	14(15.0)		
III	0(0)		
KPS score (level)		85.74 (0.45)	70–90
90 to 100	10(10.8)		
80 to 90	61(65.6)		
70 to 80	16(17.2)		
60 to 70	6(6.5)		
Time since diagnosis (months)		10.67 (1.46)	2–84
Time since the completion of previous chemotherapy (weeks)		4.28 (0.21)	1–11

SE, standard error of the mean; CIPN, chemotherapy-induced peripheral neuropathy; NSCLC, non-small cell lung cancer; SCLC, small cell lung cancer; NCI-CTCAE, The Common Terminology Criteria for Adverse Events (CTCAE) 4.03v.; KPS, Karnofsky performance score.

**Table 2:** Top ten CIPN measurements by EORTC QLQ–CIPN 20 (*N* = 93).

Variable	Domain	Mean (SE)
20. Did you have difficulty getting or maintaining an erection? (male, age < 65, *N* = 37)	Autonomic	56.02(4.33)
15. Did you have difficulty climbing stairs or getting up out of a chair because of weakness in your legs?	Motor	40.59(2.02)
17. Did you have blurred vision?	Autonomic	37.63(1.56)
16. Were you dizzy when standing up from a sitting or lying position?	Autonomic	36.56(1.60)
4. Did you have numbness in your toes or feet?	Sensory	34.95(1.84)
3. Did you have numbness in your fingers or hands?	Sensory	34.14(1.66)
11. Did you have a problem holding a pen, which made writing difficult?	Motor	30.65(1.22)
9. Did you have problems standing or walking because of difficulty feeling the ground under your feet?	Sensory	30.11(1.24)
18. Did you have difficulty hearing?	Sensory	30.11(1.30)
12. Did you have difficulty manipulating small objects with your fingers (for example, fastening small buttons)?	Motor	29.30(1.30)

EORTC QLQ-CIPN *20*, European Organization for Research and Treatment of Cancer quality of life questionnaire–chemotherapy-induced peripheral neuropathy 20, each item rated on a scale of 0–4 (not at all = 1, a little = 2, quite a bit = 3, and very much = 4), theoretical scoring range 0–100.

**Table 3:** Scores for CIPN, depression, CIPN–related QoL, and general QoL (*N* = 93).

Variable	Mean/*N*	SE/%	Range	Theoretical Scoring Range
CIPN (EORTC QLQ–CIPN 20)	31.56	0.84	0–38.89	0–100
-Sensory	29.15	0.57	0–38.89	0–100
-Motor	31.83	0.93	0–32.14	0–100
-Autonomic	33.81	1.45	0–62.50	0–100
Anxiety (HADS–anxiety subscale)	3.74	0.41	0–17	0–21
-Noncases	80.00	86.00		
-Borderline Cases	7.00	7.50		
-Clinical Cases	6.00	6.50		
Depression (HADS–depression subscale)	6.67	0.49	0–17	0–21
-Noncases	55.00	59.10		
-Borderline Cases	16.00	17.20		
-Clinical Cases	22.00	23.70		
CIPN–related QoL (FACT/GOG–Ntx)	40.44	0.57	11–44	0–44
-I have trouble feeling the shape of small objects when they are in my hand	3.88	0.46	1–4	0–4
-I have trouble hearing	3.81	0.56	1–4	0–4
-I have trouble buttoning buttons	3.80	0.58	1–4	0–4
-I have joint pain or muscle cramps	3.76	0.76	0–4	0–4
-I have trouble walking	3.75	0.72	0–4	0–4
General QoL (FACT–G)	78.08	1.68	26–102	0–108
-Physical well-being	20.79	0.59	5–28	0–28
-Social/family well-being	20.31	0.43	0–24	0–28
-Emotional well-being	19.01	0.50	1–24	0–24
-Functional well-being	17.97	0.68	3–28	0–28

CIPN, chemotherapy-induced peripheral neuropathy; QoL, quality of life; EORTC QLQ–CIPN 20, European Organization for Research and Treatment of Cancer quality of life questionnaire–chemotherapy–induced peripheral neuropathy 20, theoretical scoring range 0–100; HADS–depression subscale, Hospital Anxiety and Depression Scale, theoretical scoring range: 0–21; FACT/GOG–Ntx, The Functional Assessment of Cancer Therapy–Neurotoxicity subscale, theoretical scoring range: 0–44; FACT–G, The Functional Assessment of Cancer Therapy–General, theoretical scoring range: 0–108.

**Table 4:** Factors significantly associated with CIPN–related QoL and general QoL based on multiple regression analysis (*N* = 93).

Domains of QoL	Predictive Variable	Adjusted R^2^	Beta	F	*p*	95% CI	
						Lower	Upper
CIPN–related QoL (FACT/GOG–Ntx)	CIPN-sensory	0.645	−0.651	84.754	0.001	−2.218	−1.435
	CIPN-motor		−0.263		0.001	−1.174	−0.360
	Constant				0.001	62.024	69.974
General QoL (FACT-G)	Depression	0.756	−0.619	69.203	0.001	−2.853	−1.659
	Anxiety		−0.323		0.001	−1.943	−0.638
	Number of chemotherapy cycles		0.147		0.020	0.138	1.529
	Constant		36.051		0.001	88.355	98.725

CIPN, chemotherapy-induced peripheral neuropathy; QoL, quality of life; FACT/GOG-Ntx, The Functional Assessment of Cancer Therapy–Neurotoxicity subscale. FACT-G, The Functional Assessment of Cancer Therapy–General Input independent variable: covariates included marital status (not married vs. married), number of chemotherapy cycles (continuous score), cumulative dose of chemotherapy therapy (continuous score), CIPN–sensory (continuous score), CIPN–motor (continuous score), CIPN– autonomic (continuous score), anxiety (continuous score), and depression (continuous score).

## Data Availability

The data that support the findings of this study are available from the corresponding author. Restrictions apply to the availability of these data, which were used under license for this study. Data are available from the authors with the permission of the Chang Gung Memorial Hospital Research Program in Taiwan.
